# No Evidence That Short-Term Cognitive or Physical Training Programs or Lifestyles Are Related to Changes in White Matter Integrity in Older Adults at Risk of Dementia

**DOI:** 10.3389/fnhum.2017.00110

**Published:** 2017-03-20

**Authors:** Patrick Fissler, Hans-Peter Müller, Olivia C. Küster, Daria Laptinskaya, Franka Thurm, Alexander Woll, Thomas Elbert, Jan Kassubek, Christine A. F. von Arnim, Iris-Tatjana Kolassa

**Affiliations:** ^1^Clinical and Biological Psychology, Institute of Psychology and Education, Ulm UniversityUlm, Germany; ^2^Department of Neurology, University Hospital UlmUlm, Germany; ^3^Department of Psychology, Technische Universität DresdenDresden, Germany; ^4^Institute of Sports and Sports Science, Karlsruhe Institute of TechnologyKarlsruhe, Germany; ^5^Department of Psychology, University of KonstanzKonstanz, Germany

**Keywords:** white matter integrity, cognitive training, physical training, cognitive lifestyle, physical lifestyle, older adults, memory complaints, dementia

## Abstract

Cognitive and physical activities can benefit cognition. However, knowledge about the neurobiological mechanisms underlying these activity-induced cognitive benefits is still limited, especially with regard to the role of white matter integrity (WMI), which is affected in cognitive aging and Alzheimer’s disease. To address this knowledge gap, we investigated the immediate and long-term effects of cognitive or physical training on WMI, as well as the association between cognitive and physical lifestyles and changes in WMI over a 6-month period. Additionally, we explored whether changes in WMI underlie activity-related cognitive changes, and estimated the potential of both trainings to improve WMI by correlating training outcomes with WMI. In an observational and interventional pretest, posttest, 3-month follow-up design, we assigned 47 community-dwelling older adults at risk of dementia to 50 sessions of auditory processing and working memory training (*n* = 13), 50 sessions of cardiovascular, strength, coordination, balance and flexibility exercises (*n* = 14), or a control group (*n* = 20). We measured lifestyles trough self-reports, cognitive training skills through training performance, functional physical fitness through the Senior Fitness Test, and global cognition through a cognitive test battery. WMI was assessed via a composite score of diffusion tensor imaging-based fractional anisotropy (FA) of three regions of interest shown to be affected in aging and Alzheimer’s disease: the genu of corpus callosum, the fornix, and the hippocampal cingulum. Effects for training interventions on FA outcomes, as well as associations between lifestyles and changes in FA outcomes were not significant. Additional analyses did show associations between cognitive lifestyle and global cognitive changes at the posttest and the 3-month follow-up (β ≥ 0.40, *p* ≤ 0.02) and accounting for changes in WMI did not affect these relationships. The targeted training outcomes were related to FA scores at baseline (cognitive training skills and FA composite score, *r_s_* = 0.68, *p* = 0.05; functional physical fitness and fornix FA, *r* = 0.35, *p* = 0.03). Overall, we found no evidence of a link between short-term physical or cognitive activities and WMI changes, despite activity-related cognitive changes in older adults at risk of dementia. However, we found positive associations between the two targeted training outcomes and WMI, hinting at a potential of long-term activities to affect WMI.

## Introduction

An active cognitive and physical lifestyle can reduce the risk of cognitive decline (Valenzuela and Sachdev, 2006; Sofi et al., 2011; Ngandu et al., 2015) and dementia (Valenzuela and Sachdev, 2006; Hamer and Chida, 2009; Barnes and Yaffe, 2011). Cognitive training programs and video games showed cognitive benefits (Karbach and Verhaeghen, 2014; Lampit et al., 2014; Toril et al., 2014; Ballesteros et al., 2015), and first evidence indicated that cognitive training reduces the incidence of dementia over a 10-year period (Edwards et al., 2016). Similarly, physical activity has yielded promising results with regard to cognitive benefits (Smith et al., 2010; Nagamatsu et al., 2012; Kattenstroth et al., 2013).

Revealing the neurobiological mechanisms of the activity-induced prevention of cognitive decline and dementia could pave the way for an endogenous (Sale et al., 2014), personalized treatment approach (Cuthbert and Insel, 2013). By understanding the mechanisms of intervention effects, the identified neuropathological processes in a given patient can be targeted in an individualized fashion (Cuthbert and Insel, 2013). For example, cognitively impaired patients with deteriorated white matter integrity (WMI) may benefit more from an intervention that targets this microstructural impairment than a patient with the same behavioral syndrome but normal WMI.

However, our knowledge of the neurobiological mechanisms underlying the beneficial cognitive effects of an active lifestyle and training interventions is still in its infancy. Although there is initial evidence of functional and structural brain changes through cognitive and physical activity (Valenzuela et al., 2008, 2011; Erickson et al., 2011; Buschkuehl et al., 2012; Voss et al., 2012; Bennett D.A. et al., 2014; von Bastian and Oberauer, 2014; Constantinidis and Klingberg, 2016), the role of WMI in activity-related cognitive changes is largely unclear.

Cognitive and physical activity may increase WMI through activity-related myelination (Fields, 2015) that could lead to cognitive benefits. However, current evidence is inconsistent. While some studies support this mechanism for cognitive (Lövdén et al., 2010; Takeuchi et al., 2010; Engvig et al., 2011; Sagi et al., 2012; Steele et al., 2013; Salminen et al., 2016) and physical activities (Chaddock-Heyman et al., 2014; Svatkova et al., 2015), others do not (Voss et al., 2012; Chapman et al., 2013; Strenziok et al., 2014; Lampit et al., 2015). For example, Lampit et al. (2015) did not find cognitive training-induced changes in WMI, despite positive effects on global cognition, and Voss et al. (2012) did not observe positive effects on WMI following an extensive exercise program of three weekly 40-min sessions over the period of 1 year in a sample of 70 participants.

Moreover, there are four knowledge gaps in our understanding of physical and cognitive activity-related WMI changes. These comprise, first, training-induced WMI changes in tracts shown to be affected in cognitive aging and Alzheimer’s disease (Head et al., 2004; Ringman et al., 2007; Madden et al., 2012; Wang et al., 2012; Kantarci, 2014; Salat, 2014), second, training-induced WMI changes in a population of older adults at risk of dementia, third, maintenance of training-induced WMI changes, and fourth, lifestyle-related WMI changes.

To address the inconsistent findings and the knowledge gaps, this study had two primary aims: First, to assess the immediate and long-term effects of cognitive and physical training programs on the integrity of tracts shown to be affected in cognitive aging and Alzheimer’s disease (the genu of the corpus callosum, the fornix, and the hippocampal cingulum) in older adults at risk of dementia, and second, to investigate the relationship between cognitive and physical lifestyles and changes in WMI over the 6-month study period.

As additional analyses, we assessed the association at baseline between the two targeted training outcomes (cognitive training skills, functional physical fitness) and WMI in order to reveal the potential of training programs to affect WMI. Finally, we investigated whether changes in WMI could account for activity-related cognitive changes to understand whether changes in WMI underlie these cognitive changes.

For the cognitive training program, we used a computer-based training program targeting auditory processing and working memory that has been shown to have robust cognitive benefits (Smith et al., 2009; Zelinski et al., 2011, 2014; Bamidis et al., 2015; Shah et al., 2017). For the physical training program, we used a multimodal training regime based on a program that has previously been shown to have cognitive benefits (Thurm et al., 2011). The use of a multimodal exercise program is consistent with findings of larger cognitive benefits through combined aerobic and strength training versus aerobic exercise only (Colcombe et al., 2006; Smith et al., 2010).

With regard to our primary objectives, we hypothesized that the cognitive and physical training groups, in contrast to a passive control group, would exhibit an increase in the fractional anisotropy (FA) composite score at posttest and at the 3-month follow-up. We expected that self-reported active cognitive and physical lifestyles at baseline would be positively associated with changes in the FA composite score at both follow-ups.

## Materials and Methods

### Study Design

This 10-week interventional, two-center, controlled clinical trial (Ulm and Konstanz, Germany) entailed a three-arm assessor-blinded study evaluating training- and lifestyle-related changes in WMI. This diffusion tensor imaging (DTI) study comprises a subsample of participants of the main study whose results on the cognitive outcomes have previously been reported (Küster et al., 2016). We found that the associations of an active lifestyle with cognitive changes over time were stronger than the effects of specifically designed cognitive or physical training interventions in the same period.

### Participants

For inclusion in the study, participants had to be 55 or older, suffer from subjective memory complaints and either objective [Munich Verbal Learning Test (Ilmberger, 1988): average of the learning and free long-delayed recall trials below -1 *SD* of the age norm] or clinically apparent memory impairment (e.g., increased difficulty locating objects, keeping appointments, remembering conversations or events), have vision and hearing adjusted to normal, and be fluent in German. Exclusion criteria were a moderate or severe stage of dementia [Mini Mental State Examination (MMSE) < 20], changes in antidementive or antidepressive medication within 3 months prior to study initiation, a history of severe psychiatric or neurologic disorders, or physical impairment that would prevent participation in the physical training program. Participants without contraindications for magnet resonance imaging (MRI) were offered the opportunity to participate in the MRI subsample.

Subjects were recruited via newspaper articles, flyers, informative meetings at community centers, and personal contacts in the memory clinics of the University Hospital Ulm and the Reichenau Psychiatry Center in Konstanz. The study was approved by the Ethics Committees of the University of Konstanz and Ulm University, Germany. Participants gave written informed consent at screening visits before enrollment in the study.

**FIGURE 1:**
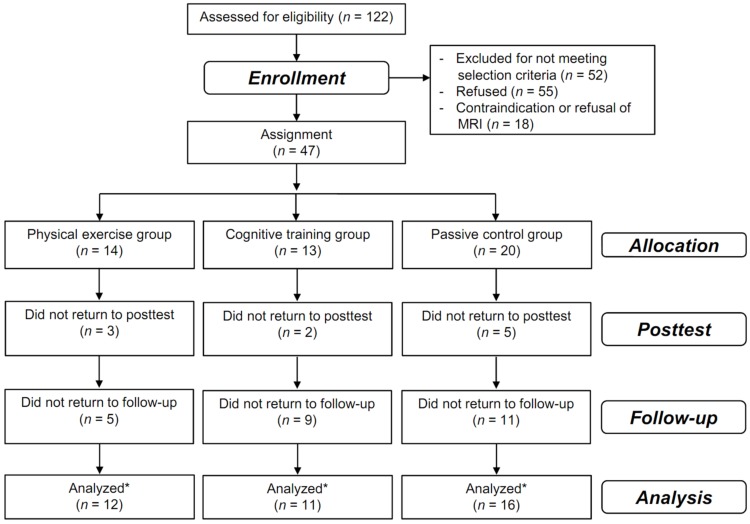
**Flow of participants.** Flow of participants within the physical training, cognitive training, and passive control groups. ^∗^all participants that were assessed at least once at the posttest or at the 3-month follow up were included in the analysis using mixed-effects models that includes all time points in a single analysis without excluding participants with missing values at one time point.

Of the 122 individuals we screened, 65 were enrolled in the intervention study (Küster et al., 2016); of these, 47 participated in the MRI subsample and were assigned to a 10-week cognitive training group (five sessions/week, *n* = 13), a physical training group (five sessions/week, *n* = 14), or a passive control group (*n* = 20, see **Figure 1**).

The analysis included 39 participants (83% of all enrolled participants). Apart from the FA of the hippocampal cingulum, the three groups did not significantly differ in terms of demographics, FA outcomes, cognitive outcomes, lifestyles, or study-related data, even without adjusting for multiple comparisons (see **Table 1**).

**Table 1:** Baseline characteristics of study groups.

Measure	Control group (*n* = 16)	Cognitive training (*n* = 11)	Physical training (*n* = 12)	*p*-value^a^
**Demographic data**				
Age, mean years ±*SD*	70.5 ± 5.0	71.4 ± 5.6	74.0 ± 5.2	0.22
Female, *n* (%)	8 (50%)	6 (55%)	9 (75%)	0.39
Education, mean years ±*SD*	15.1 ± 4.1	13.0 ± 4.2	13.9 ± 4.4	0.37
**Fractional anisotropy data**				
Composite score, mean FA ±*SD*	0.43 ± 0.04	0.44 ± 0.05	0.42 ± 0.04	0.40
gCC, mean FA ±*SD*	0.59 ± 0.05	0.58 ± 0.06	0.59 ± 0.06	0.85
Fornix, mean FA ±*SD*	0.37 ± 0.08	0.37 ± 0.06	0.33 ± 0.06	0.26
HC, mean FA ±*SD*	0.34 ± 0.05	0.38 ± 0.04	0.34 ± 0.04	0.05
**Cognitive data**				
MMSE, mean ±*SD*	28.3 ± 2.2	28.0 ± 1.7	27.8 ± 1.7	0.79
Global cognition, mean ±*SD*	-0.1 ± 1.2	0.2 ± 0.9	-0.1 ± 0.9	0.71
Executive function, mean ±*SD*	0.0 ± 1.0	0.3 ± 0.9	-0.2 ± 1.1	0.40
Episodic memory, mean ±*SD*	-0.1 ± 1.2	0.1 ± 1.0	0.1 ± 0.9	0.81
**Lifestyle data**				
Physical lifestyle, mean % ±*SD*	20.2 ± 8.8	20.3 ± 13.2	20.1 ± 9.2	>0.99
Cognitive lifestyle, mean % ±*SD*	43.3 ± 12.1	35.7 ± 15.9	36.3 ± 9.8	0.23
**Study-related data**				
Included in analysis, *n*/*n*_group_ (%)	16/20 (80%)	11/13 (85%)	12/14 (86%)	0.89


*^*a*^*p-*values of group comparisons refer to one-way ANOVA for continuous variables and to χ^*2*^ tests for categorical variables. gCC, genu of the corpus callosum; HC, hippocampal cingulum; MMSE, Mini Mental State Examination; *n*, number of participants.*

### Procedure

Outcome variables were assessed within 4 weeks before the 10-week intervention, within 4 weeks after the intervention, and another 3 months later to measure training and lifestyle-related changes in WMI. Due to logistic issues (e.g., limited available facilities, a highly selected study sample with more than 60% exclusions at screening, the required time commitment of participants, the limited time period between pretest and the start of the intervention, and the time slots of the physical training program), it was not possible to achieve the necessary number of included participants that allowed both randomized allocation and a sufficient number of participants to start a new group-based physical training program. To avoid any selection bias, the groups were matched in terms of age, education, gender, and MMSE. When a new physical training program started, all successfully screened participants were allocated to this group until the required number of participants was reached. During the other time periods, a minimization approach was implemented for the allocation of participants to the cognitive training and control groups in order to minimize group differences in age, gender, education, and MMSE. Neuropsychological outcome assessors were blind to the group allocation of participants. In rare cases, participants disclosed their group assignment during the neuropsychological assessment. The blinding of participants was not feasible due to the nature of the behavioral interventions.

### Outcomes

#### MRI Analysis

##### Data recording

The MRI analysis was performed on 1.5 Tesla scanners at the two study centers, Ulm University (center 1, Magnetom Symphony, Siemens Medical) and the University of Konstanz (center 2, Intera, Philips Medical Systems). The DTI study protocol consisted of 2 × 30 gradient directions with *b* = 1000 s/mm^2^ and two *b* = 0 gradient directions. At both centers, slice thickness was 2.5 mm and in-plane pixel size was 1.875 mm × 1.875 mm; 55 slices (128 pixels × 128 pixels) and 62 slices (128 pixels × 128 pixels) were recorded at center 1 and center 2, respectively. The echo time and repetition time were 28 and 3080 ms at center 1 and 70 ms and 8035 ms at center 2.

##### Data processing

DTI analysis was performed using the software package *Tensor Imaging and Fiber Tracking* (TIFT, Müller et al., 2007; Müller and Kassubek, 2013). For longitudinal data analysis, affine halfway linear registration (Menke et al., 2014) was employed. Pretest and posttest images were halfway-transformed, whereas follow-up images were affine transformed to the transformed pretest images. FA maps were calculated and smoothed with a Gaussian filter of 2 voxels full-width at the half maximum (FWHM, Madhyastha et al., 2014). Individualized FA templates were calculated by using FA maps of all available measurements of each individual. Based on these individualized FA templates, regions of interest (ROIs) were set. Because this processing procedure was implemented, Montreal Neurological Institute transformation was not necessary.

#### Regions of Interest

**FIGURE 2:**
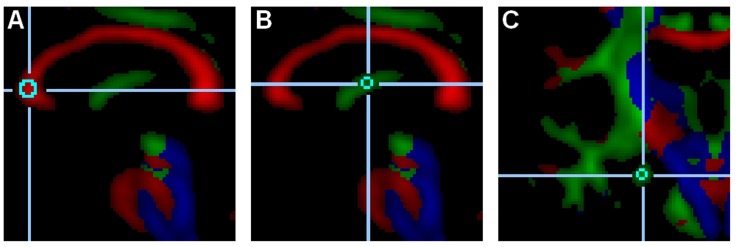
**Regions of interest.** These examples depict a 515-voxel region of interest (ROI) in the genu of the corpus callosum (**A**, midsagittal slice), a 33-voxel ROI in the fornix (**B**, midsagittal slice), and a 33-voxel ROI in the left hippocampal cingulum (**C**, coronal slice).

Regions of interests were defined in an attempt to focus on white matter correlates of cognitive aging and Alzheimer’s disease (Head et al., 2004; Ringman et al., 2007; Madden et al., 2012; Wang et al., 2012; Kantarci, 2014; Salat, 2014). To this end, the WM integrity of hippocampus-related limbic tracts and prefrontal cortex tracts were examined: the genu of the corpus callosum, the fornix and the hippocampal cingulum (see **Figure 2**). The tracts in the genu of the corpus callosum connect the two prefrontal cortices (Hofer and Frahm, 2006), and their white mater integrity has been shown to correlate with executive function (Madden et al., 2009). The fornix and the hippocampal cingulum interconnect the hippocampus with distributed brain areas; their WMI correlates with episodic memory (Bennett I.J. et al., 2014; Bennett and Stark, 2015; Ezzati et al., 2015).

Within the three ROIs (the genu of the corpus callosum, the fornix, and the hippocampal cingulum), two non-overlapping subregions were set and averaged in order to increase reliability. In the genu of the corpus callosum, the two 515-voxel subregions were set in the center of the genu of the midsagittal slice and six voxels to the right lateral direction in the center of the tract. In the fornix, the two 33-voxel subregions were set halfway between the anterior and posterior ends of the fornix in the center of the tract of the midsagittal slice and four voxels apart in the anterior-ventral direction in the center of the tract. In the hippocampal cingulum, the two 33-voxel subregions were set on the same coronal slice in the center of the tract in both hemispheres. The coronal slice was selected as the most anterior and dorsal area of the pyramidal tract. This slice – located anterior to the posterior commissure – generally cuts through the anterior pons and the midsection of the hippocampal cingulum.

The lower threshold for FA values was set to 0.2 to increase the probability that only white matter voxels would be included in the measurements (Kunimatsu et al., 2004). If fewer than 75% of all possible voxels in each subregion were above the threshold, it was lowered accordingly. Only in one participant did the threshold have to be lowered to 0.17 to include more than 75% of the fornix voxels.

#### Composite Score of WMI

A composite score of the three ROIs was constructed in order to increase statistical power by avoiding multiple comparison problems and by improving the reliability of the outcome. The composite score was calculated by averaging the FA values of the fornix, the hippocampal cingulum, and the genu of the corpus callosum.

### Cognitive Outcomes

Global cognition, episodic memory, and executive functions were assessed through an extensive cognitive test battery. Principal component analysis served to construct the three composite scores (see Küster et al., 2016). The two composite scores for episodic memory and executive function represent the weighted average of the *z*-standardized cognitive test scores with loadings of at least *a*_ij_ = 0.4 on the respective components. The global cognition score represents the average of the two component scores.

The test battery consisted of the phonemic and semantic fluency tasks, the Trail Making Test (A and B) from the CERAD neuropsychological battery (Welsh et al., 1994), the forward and backward digit span, the digit symbol coding subtest from the Wechsler Adult Intelligence Scale-III (WAIS-III, Von Aster et al., 2006), the working-memory subtest from the Everyday Cognition Battery (Allaire and Marsiske, 1999), the free recall trial from the Alzheimer’s Disease Assessment Scale – cognitive subscale (ADAS-cog, Ihl and Weyer, 1993), and the learning and free long-delayed recall trials from an adapted version of the California Verbal Learning Test (Munich Verbal Memory Test, Ilmberger, 1988).

### Interventions

#### Cognitive Training

Participants were asked to complete a total of 50 h of computerized, home-based cognitive training within a period of 10 weeks, with five 1-h sessions per week. The training consisted of six different tasks targeting auditory processing and working memory (for details see Mahncke et al., 2006a,b; Küster et al., 2016). In each session, four different 15-min training tasks were completed. The order of the tasks varied in each session; moreover, the difficulty was adapted according to the participant’s performance, and correct answers were positively reinforced. This training program was originally developed by Posit Science (San Francisco, CA, USA) and has been adapted and translated into German in a collaboration between Posit Science and the University of Konstanz. In the German version, a sound frequency discrimination task replaces the original auditory working memory task “listen and do” (see Mahncke et al., 2006b; Küster et al., 2016 for detailed training descriptions).

#### Physical Training

Participants were asked to attend a total of 20 sessions of a multimodal physical training program at the respective trial sites within a period of 10 weeks, with two 1-h sessions per week. The training was carried out in groups of 5–10 participants. In addition, a total of 30 sessions of a 20-min home-based physical training program was to be performed three times per week. These training sessions were documented by participants and monitored by the trainers. The multimodal training program involved aerobic, strength, coordination, balance, and flexibility elements and was designed in the form of an imaginary journey. The difficulty was adapted individually by the trainers to match the needs of participants. The structure of this training regime was based on a program that induced positive effects on cognition in a previous study on frail nursing-home residents (Thurm et al., 2011).

#### Passive Control Group

Wait-list control participants (controls) were asked to continue their daily life as usual and were given the opportunity to participate in one of the training programs after their follow-up assessment.

### Assessment of Lifestyle

The cognitive and physical lifestyles of participants were assessed through the Community Healthy Activities Model Program for Seniors Physical Activity Questionnaire for Older Adults (CHAMPS, Stewart et al., 2001). This questionnaire describes 40 possible activities in the participants’ daily life, categorized into physical activities (such as running, swimming, or bicycling) and cognitively challenging activities (such as playing card or board games, performing voluntary work, or playing a musical instrument; see Küster et al., 2016). Participants were asked to report the activities in which they had engaged in the previous four weeks. The number of completed activities was divided by the potential number of activities in each domain. These scores reflect the variety in the participants’ cognitive and physical lifestyles, respectively.

### Cognitive Training Skills

Cognitive training skills were measured by averaging the standardized training performance in the most frequently used cognitive training tasks: “high or low,” “tell us apart,” “sound replay,” and “match it.” Changes in cognitive training skills were measured in terms of the difference between the third and the last training session (the first two training sessions were guided by trainers). Unfortunately, the cognitive training data from two individuals were not properly stored and could not be included in the analysis.

### Functional Physical Fitness

Functional physical fitness was assessed with four tasks from the Senior Fitness Test (Rikli and Jones, 2001): “chair stand,” “chair sit-and-reach,” “2-min step,” and “8-feet up-and-go” which measure strength, flexibility, endurance, and agility, respectively. *Z*-standardized scores were averaged to create the functional physical fitness composite score.

### Statistical Analyses

Statistical analyses were conducted using *R* version 3.2.1 for Windows (R Development Core Team, 2015). To assess baseline differences between the three groups, χ^2^-tests and one-way analyses of variance were used for categorical variables and continuous variables, respectively.

#### Training- and Lifestyle-Related FA and Cognitive Changes

The effects of training interventions on WMI as well as lifestyle-related changes in WMI were assessed with linear mixed-effects models with maximum likelihood estimation (nlme package, Pinheiro et al., 2000). Group (with contrasts cognitive training vs. controls and physical training vs. controls), physical lifestyle, cognitive lifestyle, and time (with contrasts pre vs. post and pre vs. follow-up) were defined as fixed effects, and subject as the random intercept. Hypothesis-relevant effects were indicated by Group × Time, Physical Lifestyle × Time, and Cognitive Lifestyle × Time interactions. Hedges’ *g* was based on the pretest standard deviation; this was calculated by the difference in change scores between (1) the physical training group vs. the control group and (2) the cognitive training group vs. the control group divided by the pooled baseline standard deviation corrected for bias in small samples (Lakens, 2013). Positive values indicate beneficial effects of the intervention. Standardized regression coefficients of cognitive and physical lifestyles predicting changes in outcomes were used as effect size measure for lifestyle-related outcome changes.

#### The Potential of the Two Training Programs to Affect White Matter Integrity

To assess the potential of the cognitive and physical training programs to improve hippocampus-related and prefrontal WMI, we performed two analyses: (1) at pretest, we assessed the cross-sectional correlations of cognitive training skills and functional physical fitness with FA and cognitive outcomes, and (2) we investigated the improvement in cognitive training skills and functional physical fitness within the respective training groups. For the analyses of cognitive training skills, we used non-parametric procedures (Spearman’s rank correlation and Wilcoxon signed rank test for paired differences) due to the small sample size (*n* = 9).

#### Reliability of FA Scores

Retest-reliability was assessed through correlations between pretest and posttest scores within the total study sample including all three groups.

## Results

### Effects of Cognitive and Physical Training on WMI and Cognition

We did not find a significant influence of the cognitive or physical training program on WMI compared to the control group, neither at the posttest (all *p*s ≥ 0.18 before adjustment of multiple comparisons; Hedges’ *g*s ≤ 0.25) nor at the 3-month follow-up (all *p*s ≥ 0.16; Hedges’ *g*s ≤ 0.31). Hedges’ *g*s of the FA composite score were -0.09, 95% CI [-0.43, 0.22] at posttest and -0.14, 95% CI [-0.90, 0.57] at the 3-month follow-up for the cognitive training, and 0.03, 95% CI [-0.41, 0.47] at posttest and -0.18, 95% CI [-0.79, 0.40] at the 3-month follow-up for the physical training (see **Table 2**).

**Table 2:** Effects of training interventions.

Measure	Control group (*n* = 16)	Cognitive training (*n* = 11)			Physical training (*n* = 12)		
			
Time point	Change (95% CI)	Change (95% CI)	*p*^a^	*g^b^*	Change (95% CI)	*p*^a^	*g^b^*
**Composite scores**			
FA composite							
Posttest	0.0004 (-0.010 to 0.011)	-0.004 (-0.012 to 0.004)	0.57	-0.09	0.001 (-0.013 to 0.016)	0.85	0.03
3-month FU	0.0001 (-0.018 to 0.018)	-0.006 (-0.039 to 0.026)	0.41	-0.14	-0.007 (-0.023 to 0.008)	0.49	-0.18
Global cognition							
Posttest	0.53 (0.34 to 0.72)	0.35 (0.06 to 0.65)	0.32	-0.16	0.23 (-0.07 to 0.53)	0.09	-0.27
3-month FU	1.28 (1.00 to 1.56)	0.91 (0.00 to 1.81)	0.12	-0.33	1.14 (0.63 to 1.65)	0.61	-0.12
**Specific scores**							
gCC							
Posttest	0.013 (0.001 to 0.025)	-0.001 (-0.011 to 0.009)	0.18	-0.25	0.002 (-0.019 to 0.024)	0.45	-0.19
3-month FU	0.000 (-0.016 to 0.016)	0.013 (-0.001 to 0.027)	0.50	0.22	-0.017 (-0.043 to 0.009)	0.16	-0.29
Fornix							
Posttest	-0.018 (-0.034 to -0.002)	-0.003 (-0.022 to 0.016)	0.23	0.21	0.001 (-0.018 to 0.019)	0.18	0.25
3-month FU	-0.008 (-0.042 to 0.027)	-0.030 (-0.073 to 0.013)	0.17	-0.29	0.016 (-0.017 to 0.049)	0.25	0.31
HC							
Posttest	0.006 (-0.011 to 0.024)	-0.007 (-0.018 to 0.004)	0.23	-0.28	0.001 (-0.022 to 0.024)	0.58	-0.11
3-month FU	0.008 (-0.020 to 0.036)	-0.003 (-0.063 to 0.058)	0.54	-0.20	-0.020 (-0.038 to -0.003)	0.18	-0.59
Executive function							
Posttest	0.39 (0.14 to 0.65)	0.20 (-0.00 to 0.41)	0.34	-0.19	0.30 (-0.09 to 0.69)	0.63	-0.09
3-month FU	0.39 (-0.01 to 0.79)	-0.09 (-0.86 to 0.67)	0.04	-0.46	0.35 (-0.23 to 0.94)	0.65	-0.03
Episodic memory							
Posttest	0.54 (0.28 to 0.81)	0.41 (0.01 to 0.82)	0.62	-0.12	0.12 (-0.27 to 0.52)	0.10	-0.39
3-month FU	1.82 (1.40 to 2.24)	1.62 (0.23 to 3.01)	0.64	-0.17	1.61 (0.86 to 2.37)	0.70	-0.18


*^*a*^*p-*value of the Group [Control vs. Cognitive/Physical Training] × Session [pre vs. post and pre vs. 3-month FU] interaction, before adjustment for multiple comparisons. ^*b*^Hedges’ *g*: change in cognitive/physical training minus change in control group divided by the pooled baseline standard deviation corrected for bias in small samples. Positive values indicate beneficial effects of the interventions. gCC, genu of the corpus callosum; FA, fractional anisotropy; FU, follow-up; FX, fornix; HC, hippocampal cingulum; MMSE, Mini Mental State Examination; *n*, number of participants.*

Likewise, we did not find a significant impact of both training programs on global cognition compared to the control group, neither at the posttest (all *p*s ≥ 0.09; Hedges’ *g*s ≤-0.16) nor at the 3-month follow-up (all *p*s ≥ 0.12; Hedges’ *g*s ≤ -0.12; see **Table 2**).

### Cognitive and Physical Lifestyle-Related Changes in WMI and Cognition

We did not find significant associations between self-reported cognitive and physical lifestyles at baseline and changes in WMI, neither at the posttest (all *p*s ≥ 0.08 before adjustment of multiple comparisons; all βs ≤ 0.34) nor at the 3-month follow-up (all *p*s ≥ 0.31 before adjustment of multiple comparisons; all βs ≤ 0.20). Effect sizes for the FA composite score were β = 0.20, 95% CI [-0.16, 0.56] at the posttest and β = -0.04, 95% CI [-0.54, 0.45] at the 3-month follow-up with respect to cognitive lifestyle, and β = -0.04, 95% CI [-0.40, 0.32] at the posttest and β = 0.15, 95% CI [-0.34, 0.64] at the 3-month follow-up with respect to physical lifestyle (see **Table 3**).

**Table 3:** Associations with cognitive and physical lifestyles.

Measures	Cognitive lifestyle (*n* = 39)		Physical lifestyle (*n* = 39)	
		
Time point	β^a^ (95% CI)	*p-*value^b^	β^a^ (95% CI)	*p-*value^c^
**Composite scores**			
FA composite				
Posttest	0.20 (-0.16 to 0.56)	0.27	-0.04 (-0.40 to 0.32)	0.80
3-month FU	-0.04 (-0.54 to 0.45)	0.97	0.15 (-0.34 to 0.64)	0.69
Global cognition				
Posttest	0.51 (0.23 to 0.80)	0.004	0.12 (-0.17 to 0.41)	0.49
3-month FU	0.40 (-0.01 to 0.82)	0.01	0.12 (-0.29 to 0.54)	0.71
**Specific scores**			
gCC				
Posttest	0.34 (-0.00 to 0.69)	0.08	0.02 (-0.32 to 0.36)	0.92
3-month FU	-0.20 (-0.68 to 0.29)	0.31	0.12 (-0.37 to 0.61)	0.71
Fornix				
Posttest	-0.10 (-0.46 to 0.26)	0.63	0.19 (-0.17 to 0.55)	0.36
3-month FU	-0.06 (-0.56 to 0.43)	0.95	0.15 (-0.34 to 0.64)	0.74
HC				
Posttest	0.18 (-0.17 to 0.53)	0.30	-0.28 (-0.64 to 0.07)	0.13
3-month FU	0.16 (-0.32 to 0.66)	0.33	-0.01 (-0.50 to 0.48)	0.92
Executive function				
Posttest	0.26 (-0.08 to 0.60)	0.16	-0.08 (-0.42 to 0.26)	0.67
3-month FU	0.08 (-0.39 to 0.54)	0.21	-0.02 (-0.49 to 0.44)	0.77
Episodic memory				
Posttest	0.45 (0.15 to 0.74)	0.02	0.21 (-0.09 to 0.50)	0.28
3-month FU	0.41 (0.00 to 0.82)	0.02	0.16 (-0.25 to 0.57)	0.57


*^*a*^β*s* represent the standardized regression coefficients of cognitive and physical lifestyles predicting changes in outcomes. ^*b*^*p*-value of the Cognitive Lifestyle × Session [pre vs. post and pre vs. 3-month FU] interaction. ^*c*^*p*-value of the Physical Lifestyle × Session [pre vs. post and pre vs. 3-month FU] interaction. gCC, genu of the corpus callosum; FA, fractional anisotropy; FU, follow-up; FX, fornix; HC, hippocampal cingulum; MMSE, Mini Mental State Examination; *n*, number of participants.*

**FIGURE 3:**
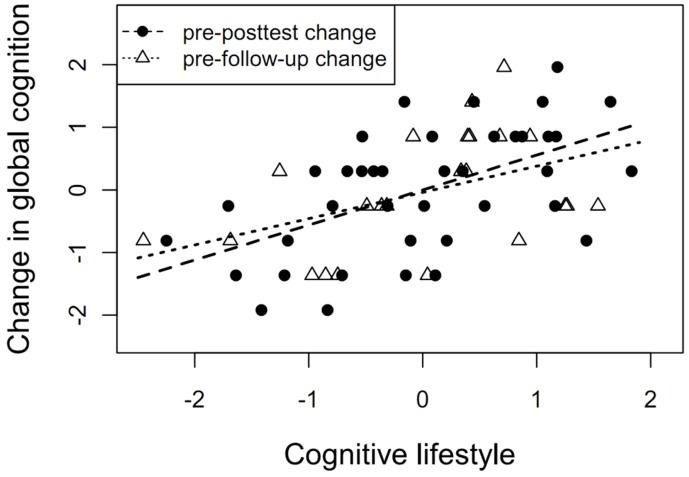
**Cognitive lifestyle as a predictor of cognitive change.** Association between self-reported cognitive lifestyle at baseline and changes in global cognition from pretest to posttest (β = 0.51, *p* = 0.004) and from pretest to 3-month follow-up (β = 0.40, *p* = 0.01).

Despite the lack of significant lifestyle-related FA changes, we found an association between cognitive lifestyle and changes in both global cognition and episodic memory from the pretest to the posttest and to the 3-month follow-up (all *p*s ≤ 0.02, all βs ≥ 0.40; see **Figure 3** and **Table 3**).

### Additional Analyses

#### The Potential of the Two Training Programs to Affect White Matter Integrity

**FIGURE 4:**
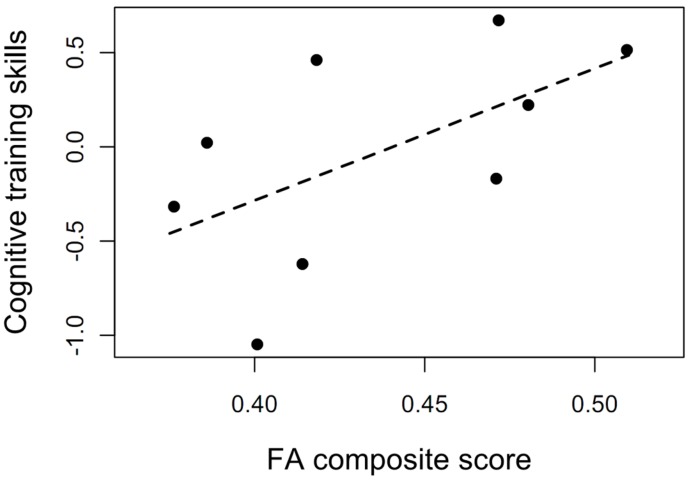
**The potential of the cognitive training program to affect white matter integrity.** Association between cognitive training skills at the beginning of the training and FA composite score at baseline (*r_s_* = 0.68, *p* = 0.05). The cognitive training data from two individuals were not properly stored and could not be included in the analysis.

Additional analyses showed that cognitive training skills at the start of the program were correlated with the FA composite score, *r_s_* = 0.68, *p* = 0.05, indicating the potential of the cognitive training program to affect WMI and the fact that engagement in cognitive training taps the neural connections of interest (see **Figure 4**). Associations between the various ROIs and the cognitive training skills were similar, with medium to large effect sizes: fornix, *r_s_* = 0.50, *p* = 0.18; hippocampal cingulum, *r_s_* = 0.33, *p* = 0.39; genu of the corpus callosum, *r_s_* = 0.60; *p* = 0.01.

In the cognitive training group, we found a significant increase in cognitive training skills over the training period, with a very large effect size, *g* = 1.68, *p* = 0.008. Performance changes in all four training tasks revealed medium to very large effect sizes: “match it,” *g* = 1.47, *p* = 0.02; “sound replay,” *g* = 0.52, *p* = 0.20; “high or low,” *g* = 0.89, *p* = 0.008; “tell us apart,” *g* = 0.95, *p* = 0.10.

**FIGURE 5:**
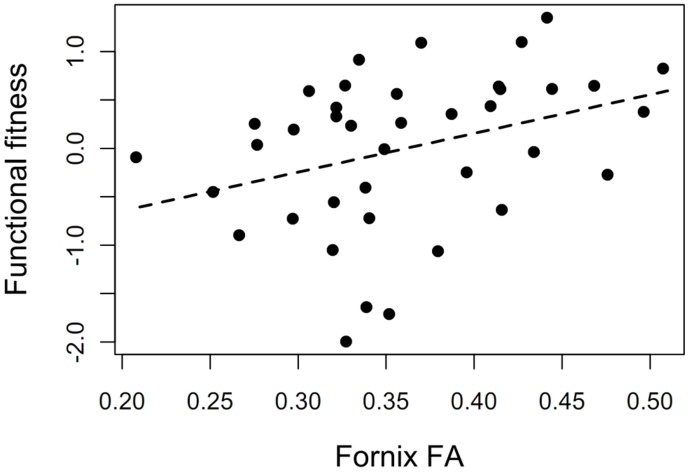
**The potential of the physical training program to affect white matter integrity.** Association between functional physical fitness and fornix FA at baseline (*r* = 0.35, *p* = 0.03).

Functional physical fitness was marginally significantly associated with the FA composite score, *r* = 0.28, *p* = 0.08, and significantly related to the fornix FA, *r* = 0.35, *p* = 0.03 (see **Figure 5**) indicating that interventions that target physical fitness have the potential to affect WMI.

In the physical training group, we found a significant increase in functional physical fitness over the study period, *p* = 0.02. This increase was marginally significant at the posttest, β = 0.51, *p* = 0.07, and significant at the 3-month follow-up, β = 0.88, *p* = 0.007.

#### Associations between Changes in Targeted Training Outcomes and FA Changes

Changes in cognitive training skills were not associated with changes in the FA composite score, *r_s_* = -0.27, *p* = 0.49, or in global cognition, *r_s_* = 0.20, *p* = 0.61. Likewise, changes in functional physical fitness did not correlate with changes in the FA composite score at posttest, *r* = -0.19, *p* = 0.28, or at follow-up, *r* = 0.01, *p* = 0.96, nor in global cognition at posttest, *r* = -0.10, *p* = 0.58, or at follow-up, *r* = -0.14, *p* = 0.54.

#### Reliability of FA Measures

Retest-reliability between pretest and posttest was high for the composite FA score, *r* = 0.91, and ranged from *r* = 0.92 for the genu of the corpus callosum to *r* = 0.91 for the fornix and *r* = 0.80 for the hippocampal cingulum.

## Discussion

We found no evidence of an effect of short-term cognitive or physical training programs on WMI in regions that have previously been shown to be affected in cognitive aging and Alzheimer’s disease (the genu of the corpus callosum, the fornix, and the hippocampal cingulum) in a sample of older adults at risk of dementia (Head et al., 2004; Ringman et al., 2007; Madden et al., 2012; Wang et al., 2012; Kantarci, 2014; Salat, 2014). The estimated effect sizes of the two training programs at the posttest were not of relevance (Hedges’ *g* < 0.1), and the two 95% confidence intervals did not include medium effects (Hedges’ *g* < 0.5).

The lack of training-induced changes in FA is consistent with several previous findings. For example, for the cognitive training program used in our study, Strenziok et al. (2014) did not find any effect on FA scores compared to two other video games. Moreover, in one of the largest studies in the field, Voss et al. (2012) did not show significant FA increases in a 1-year aerobic fitness training intervention compared to a stretching control intervention.

It is worth to note that physical training has been shown to increase FA in fiber tracts implicated in motor functioning such as the corticospinal tract (Svatkova et al., 2015). These tracts were not of interest in this study and potential effects could not be detected in our ROI analysis.

The lack of a cognitive training effect contrasts with three studies that found significant effects of different working memory training programs on regions of the anterior part of the corpus callosum (Lövdén et al., 2010; Takeuchi et al., 2010; Salminen et al., 2016). These inconsistent results might be explained by the working memory training and by the study population. In contrast to the other studies our working memory training did not include an updating component and our sample comprised older adults at risk of dementia vs. younger adults in Takeuchi et al. (2010) and Salminen et al. (2016), and healthy older adults in Lövdén et al. (2010).

The associations between cognitive training skills and the FA composite, as well as between functional physical fitness with the fornix FA hint at the potential of cognitive and physical activities to improve WMI in these tracts. Correlations between these two training outcomes and FA transfer outcomes allow us to estimate the maximal transfer gains given a specific increase in the training outcomes (Jaeggi et al., 2010; Baniqued et al., 2013; Rode et al., 2014). The higher the association, the higher is the transfer potential. Therefore, long-term rather than short-term training programs and lifestyles that induce larger effects on training outcomes may significantly increase the targeted white matter tracts.

Self-reported lifestyles at baseline were not associated with changes in WMI. In addition, positive associations between cognitive lifestyle and changes in global cognition and episodic memory were not altered after accounting for WMI. To our knowledge, there has been no other study that has assessed the relationship between lifestyles and changes in WMI. Therefore, this is initial evidence that other brain mechanisms than changes in WMI do underlie lifestyle-related cognitive changes in older adults at risk of dementia.

### Limitations

Our use of ROI analyses rather than whole brain-based approaches means that any changes in other brain regions would not be detected. However, in this sample of older adults at risk of dementia, we were particularly interested in the white matter tracts that are affected in cognitive aging and Alzheimer’s disease. Importantly, by using ROIs, we limited the problems of alpha-error inflation and a reduction in power through multiple comparisons – an issue that is particularly important in analyses with limited sample sizes. Other limitations include the lack of randomization, which was not feasible due to logistic issues (see above). However, we used a minimization approach instead to prevent group differences in participants’ characteristics from inducing bias. The limited sample size likely impeded the detection of very small effects. However, the sample size was sufficient to detect lifestyle-related cognitive changes and to reveal associations between WMI and both cognitive training skills and functional physical fitness. In addition, the confidence intervals of the training effects immediately after the training period were lower than a Hedges’ *g* of 0.5, suggesting that effects of medium size are unlikely. Finally, the lack of a lifestyle intervention prevented a causal inference regarding associations between lifestyles and FA changes. However, before implementing cost-intensive experimental designs, it is a reasonable strategy to initially employ observational designs.

### Future Perspectives

Future studies should use larger samples to increase the probability of finding small effect sizes; moreover, they should lengthen the training periods to enhance the potential to induce larger effects. In addition, little is known about the time course and maintenance of activity-induced white matter changes, suggesting that future studies should implement multiple assessments during the training regime and after the training period. Activity-related white matter changes may be differential for specific populations; thus, younger participants without cognitive impairments may profit more than older adults at risk of dementia. Future meta-analyses should assess these potential moderators. Interventional studies have only rarely reported the correlation of training outcomes with potential neurobiological mechanisms and have neglected the relation between cognitive and neurobiological changes. Future interventional studies should include these analyses to allow a better understanding of the mediating role of WMI for cognitive benefits. Finally, to our knowledge, cognitive and physical lifestyle-related changes in WMI have not yet been reported. Large-scale studies investigating this association should be conducted as a first step to explore the role of active cognitive and physical lifestyles for WMI.

### Conclusion

First, we found no evidence that short-term cognitive and physical training programs do affect the integrity of hippocampus-related and prefrontal white matter tracts in older adults at risk of dementia. Second, we provide first evidence that WMI changes do not underlie the positive association between a cognitive lifestyle and cognitive change. However, as the two training outcomes (cognitive training skills and functional physical fitness) were related to WMI, engagement in long-term cognitive and physical activities might have the potential to affect WMI.

## Author Contributions

PF contributed to study conception and design, organized study procedures and acquired data, analyzed and interpreted data, and wrote the first draft of the manuscript as well as the paper. H-PM designed the MRI protocol, supervised the DTI analysis, was involved in the interpretation of the imaging results, and critically revised the manuscript for intellectual content. OK and DL contributed to study conception and design, organized study procedures and acquired data, contributed to the data analysis and interpretation of results, and critically revised the manuscript for intellectual content. FT contributed to study conception and design, organized study procedures, acquired data, and critically revised the manuscript for intellectual content. AW designed the physical training program and revised the manuscript. TE contributed to study conception and design and critically revised the manuscript for intellectual content. JK designed the MRI protocol, supervised the DTI analysis, was involved in the interpretation of the imaging results, and critically revised the manuscript for intellectual content. CvA and I-TK conceptualized the study, obtained funding, supervised all phases of the study as principle investigators, and critically revised the manuscript for intellectual content. All authors read and approved the final manuscript.

## Conflict of Interest Statement

TE and I-TK are members of the scientific advisory board of Posit Science. CvA received honoraria from serving on the scientific advisory board of Nutricia GmbH and Honkong University Research council, travel funding and speaker honoraria from Nutricia GmbH, Novartis Pharma GmbH, Lilly Deutschland GmbH, Desitin Arzneimittel GmbH, and Dr. Willmar Schwabe GmbH & Co. KG, and research support from Roche Diagnostics GmbH, Biologische Heilmittel Heel GmbH, and ViaMed GmbH. The other authors declare that the research was conducted in the absence of any commercial or financial relationships that could be construed as a potential conflict of interest.

## Availability of Data and Materials

Data will be made available upon request. The participants did not approve the unrestricted publication of the data in their informed consents, as this option was not common at the time.

## Ethics statement

This study was carried out in accordance with the recommendations of the Ethics Committees of the University of Konstanz and Ulm University with written informed consent from all subjects. All subjects gave written informed consent in accordance with the Declaration of Helsinki. The protocol was approved by the Ethics Committees of the University of Konstanz and Ulm University, Germany.
